# Deciding upon Transition to Residential Care for Persons Living with Dementia: why Do Iranian Family Caregivers Living in Sweden Cease Caregiving at Home?

**DOI:** 10.1007/s10823-017-9337-1

**Published:** 2017-11-23

**Authors:** Mahin Kiwi, Lars-Christer Hydén, Eleonor Antelius

**Affiliations:** Centre for Dementia Research (CEDER), Division Ageing & Social Change (ASC), Department of Social and Welfare Studies, Linköping University, CEDER/ISV, 601 74 Norrköping, Sweden

**Keywords:** Caregiver, Dementia diseases, Iranian immigrants, Care at home, Care at residential nursing home, Transition

## Abstract

Previous research has shown how filial piety is strong among people of Iranian background and that traditional Iranian culture result in most families’ preferring to care for their elderly (and sick) family members at home. While acknowledging this, this article highlights what living in diaspora could mean in terms of cultural adaption and changing family values. By interviewing people with Iranian background living in Sweden (*n =* 20), whom all have been former primary caregivers to a relative living with dementia, we are able to show how the decision to cease caregiving at home is taken, and what underlying factors form the basis for such decision. Results indicate that although the existence of a Persian profiled dementia care facility is crucial in the making of the decision, it is the feeling of ‘sheer exhaustion’ that is the main factor for ceasing care at home. And, we argue, the ability to make such a decision based upon ‘being too tired’ must be understood in relation to transition processes and changes in lifestyle having an affect upon cultural values in relation to filial piety. Because, at the same time the changes on cultural values might not change accordingly among the elderly who are the ones moving into residential care, resulting in them quite often being left out of the actual decision.

## Introduction

Dementia is a chronic neurodegenerative disease mainly associated with aging and as more and more people tend to live longer, more and more people will become affected. Several studies have shown that dementia affected about 44.4 million people worldwide in 2013 with an expected increase of 75.6 million people by 2030 (Ngo and Holroyd-Leduc 2014). A systematic review of recent dementia practice guidelines (Ngo and Holroyd-Leduc 2014) has further indicated that the increase in the number of persons with dementia is higher among immigrant populations from developing countries living in western countries (also cf. Ferri et al. 2006). As the disease increases among migrant populations, the challenges about how to organize care for immigrant populations with dementia become increasingly pressing. At the same time knowledge from clinical practice is lacking (Priebe et al. 2011).

This study specifically examines persons within the Iranian immigrant population in Sweden who has a dementia diagnosis. The difficulties encountered by the Iranian migrant population in addressing the problems related to dementia stems from several factors. The problem is worsened for immigrant populations in western countries to care for their aging ones at home (care for people at home is referred as family care) (Omeri 1997). Omeri (1997:15) believes that Iranians reluctance to accept formal, institutional care may be due to cultural differences. Iranians’ care have “specific meanings, experiences, and expressions,” and that can be lost when they receive formal care. Omeri (1997) has suggested in her study of the Iranian population in Australia, that differences in cultural beliefs, values and cultural meanings that are associated with expressions of health and illness are central to quality and culturally specific and meaningful care.

Several scholars strongly argue that the discussion and analysis of migrant populations, such as Iranian population in Sweden cannot be made without reference to why they immigrated to their present countries of residence (Emami and Ekman 1998; Emami and Torres 2005; Emami 2000; Hajighasemi 1994; Torres 2001, 2006). In seeking to understand why migrant populations immigrated to their host nations, some scholars suggest that the increase in migration is caused by global interconnectedness and globalization (Castles 2002). However, it is also noted that migration in general is an extremely complex phenomenon that changes over time and its causes cannot be entirely attributed to the forces of globalization. Emami and Ekman (1998) and Hajighasemi (1994) both argue that the main reason why older people emigrate is to be united with their children and be cared for by them. Regarding elderly Iranians, according to Emami and Ekman (1998), the reason that some of the older Iranian immigrants came to Sweden is due to lack of institutional care for old people in Iran. Even though many care services such as home care services are available in Sweden, the close family ties within Iranian families leads to relative’s caregiving to a great extent (Hajighasemi 1994). However, the more the children and grandchildren adapt to Swedish society, the less the families will have time to care for their old ones (ibid; Kiwi, Iranian relative's attitudes towards culturally profiled nursing homes for individuals living with dementia, unpublished). Consequently, children that are already integrated into society do not see it as their duty to care for their older relatives (Emami and Ekman 1998).

Today there exist many different types of (culturally) profiled nursing homes in Sweden. Currently, the experience of transition from caregiving within the family to care at an ethnoculturally profiled nursing home with caregivers with Iranian origin in Sweden is unknown. Hence, the aim of this study is to explore the family care givers’ decision to cease caregiving at home and move their family members with dementia to a culturally profiled nursing home in Sweden.^1^

## Iranian Family Caregiving

Some Iranians believe the family has a major responsibility to take care of their elderly and less fortunate family members and thus a person without immediate family members is considered joyless and has no future. Within the family network, people have their own roles and duties towards the elderly. Taking care of their elderly is a status marker in itself, and strengthens the network of family prestige in friend and family circles. Religion in general (including Islam) appreciates the elderly and thus conveys the message that young people should treat older people well, show them respect, and even support them. Although religion is woven into many individuals’ character, and despite the traditional view of the family and the elderly, many older people have been caught in difficult situations. Many sociologists and social psychologists have noted elderly social problems form as a result of loneliness, and they now write about the problem (Riahi 2008; Heravi-Karimooi et al. 2010).

### Iranians in Sweden

Before the revolution in of 1979 in Iran, Sweden had few Iranian immigrants (approx. 1000) of whom most were students. However, due to the revolution and following years of warfare between Iraq and Iran many chose/had to leave. During this first exodus, nearly 2200 Iranians were granted asylum in Sweden, a number which has increased greatly since then. Within ten years there were more than 17,000 Iranians living in Sweden and according to a census taken in 2013, the Iranian population numbered 67,211 (SCB 2016). According to recent data from statistics Sweden, Iranians are now the fifth largest immigrant group residing in Sweden (SCB 2016) and of the 10 largest immigrant groups in Sweden, Swedish-Iranians has one of the lowest un-employment rates as well as the highest proportion of college/university students.

Many of the Iranians who moved to Sweden have tried to bring their parents to the country, but have faced many problems (Emami 2000; Hajighasemi 1994). Research has shown that family reunification does not necessarily mean that a person ceases to be alone or in need, and proximity to the children in Sweden is not always successful (ibid). This could be as a result of diversity and heterogeneity in terms of social class, age, education, and religion. It should be noted that many of the older Iranians brought to Sweden by their relatives (family members) were not previously exposed to western cultural life styles. In this regard, they have been finding it difficult to adjust to life in Sweden. For instance, the previously strong desire and sense of filial piety to take care of the elderly might have decreased to a higher degree among the younger living in Sweden, but not to the same extent among the elderly coming from Iran. A phenomenon sometimes described as the children’s “acculturation” into a different society. Further, there are presently no statistics on how many elderly Iranians who now resides in Sweden who have been diagnosed with dementia, or what form of care they use, but as will be discuss in the results section below, most of the persons with dementia included in this study have been cared for in their homes either solely by relatives or by relatives in combination of utilizing home help care before moving into residential care.

### Swedish Long-Term Care System

Contrary to the more traditional Iranian care system, which is or has been more family-care based (see for instance Abdollahpour et al. 2012; Hajighasemi 1994) the Swedish long-term care system is welfare state based and most Swedes rely on the state to take care of the elderly.^2^ It is a system based upon the philosophical aim of providing the elderly support to live an independent life of high quality. Before the 1990s most of this care was catered for in institutions but for both financial and philosophical reasons, it is nowadays considered beneficial to stay as long as possible in one’s own home, something which has come to be known as the *Stay-at-home-policy*. This means then, that the prevailing option is that for most individuals it is considered better to age in place, with proper/necessary support (such as home help care, meal services, safety alarms, home adaptation, transportation services etc.) and avoid relocation to residential homes as far as possible (see for instance Söderberg et al. 2013). Residential care is not un-common among the oldest old even if the numbers are dropping also within that age group. In 2013, 12,9% of those aged 80+ lived within residential care, which could be compared with for instance 30% in 1975 (Fukushima et al. 2010; SoS 2015). New studies also show that a move to residential care often is preceded by a hospital stay and many do not stay long; just over two in ten will die within six months and more than four out of ten within a year and a half (SoS 2015).

In an attempt to theorize relocation and the factors that affect the institutionalization process Luppaa et al. (2008) argues that predisposing-, need-, and enabling variables all are important to predict institutionalization in dementia. Although Luppaa and colleagues highlight both primary and secondary stressors, these concepts do not conceptualize the specific family process leading to institutionalization. Instead, we argue that the concepts introduced by Hirschfeld (1979) which later on has been explicated by McGrew (1995) allow a better understanding of the family processes that are involved in coping with family stressors and conflicts, and making decisions about moving elderly family members to a nursing home.

Hirschfeld (1979, 2003) identified three factors – capacity, tension and mutuality – that all contribute to motivate family caregiving and nursing home placement of the family member with dementia. Capacity is the ability of the caregiver to exploit and spend his/her reserves. The steeper the decline of the care recipient’s health, the more reserves must be spent, and the less able the caregiver is to achieve objectives. The concept tension refers to negative attitudes and obstacles between a caregiver and the person with dementia, and especially denotes the caregiver’s number of unmet needs in combination with their ascribed importance. Finally, mutuality between supportive and family members with dementia grows out of a caregiver’s desire to find gratification in their relationship as a care-giver with the diseased. An important factor in mutuality is the caregiver’s ability to find a reciprocation of the care given in whether or not the diseased individual perceives them.

These concepts, which has also been used by McGrew (1995) to show different patterns and perspectives in caregiving trajectories, will prove helpful in analyzing and understanding how relatives reason and argue around making decisions about moving family members with dementia to nursing homes.

## Method, Material and Data Analysis

### Setting

Culturally profiled dementia care facilities are a quite recent, very specific, care form in Sweden and as such the care home which is central to the argument in this article is the only Persian one existing in Sweden at the time of the study (there are however other profiles at other care facilities such as Middle Eastern, Oriental, Jewish, or Finnish).^3^ This study is based on interviews with family members to residents at such a residential dementia care facility in Sweden with a Persian focus. This includes not just the ability to speak Persian with the staff but also that everything about it should remind the residents of “being home in Iran”. Hence, furniture, carpets, paintings, smells, food etc. are all chosen in order to create this feeling of “being home”. Most of the staff (nurses, assistant nurses and doctors) are Persian speakers, some of them multilingual (speaking Swedish as well as other languages such as Arabic, Azerbaijani and Kurdish). There is also a chef who prepares culturally appropriate food. The residents all have their own rooms and each room has a kitchen, toilet, bathroom, and balcony. There is also a common area which all have access to and where most meals are taken and where there is a TV and sitting groups etc.

In total, 17 individuals with a dementia diagnosis lived at the studied unit, with in total 20 relatives who had had the primary role as a previous home caregiver and the official decision maker for the person with dementia. This group included one trustee as the person with dementia lacked present relatives. In total, all these 20 primary family caregivers were approached for participation in the study, and all accepted. Participants were all of Iranian origin, had lived in Sweden between 8 to 34 years; were between 30 to 63 years of age; 11 female and nine male; nine daughters, eight sons and two wifes and one trustee (Table 1).

**Table 1:** Sample characteristics’ of persons interviewed and person with dementia (PWD)

Gender	Age	Duration of stay in Sweden (Years)	Relation to the PWD	Duration of stay in Sweden for the PWD (year)	Length of time of diagnosis prior to moving to the residential home
Female	45	31	daughter	31	2 Years
F	40	26	daughter	25	4 Years
F	47	30	daughter	26	1½ months
F	59	18	daughter	15	2 Years
F	61	29	daughter	18	5 Years
Male	55	27	son	18	1 month
M	59	29	son	10	4 Years
M	62	36	son	29	3½ years
M	37	15	son	8	1 month
F	57	29	daughter	19	Unknown
M	63	35	son	24	6 months
F	45	29	daughter	19	7 years
M	62	30	trustee	33	1 week after
F	50	26	daughter	20	2 years
F	73	34	wife	34	Unknown
F	58	35	daughter	32	3 years
F	54	25	wife	25	1 year
M	63	30	son	29	Unknown
M	30	Born in Sweden	son	34	Unknown
M	37	32	son	32	Unknown

Semi-structured interviews were conducted between spring and autumn in 2013 at the nursing home. An interview guide was constructed (appendix 1) prior to data collection based on findings from author MK’s previous 1 year-long field study of said nursing home. It thus contained the following themes that had proven to be of interest while conducting the participant observations: effect of caregiving on quality of life, reason for the choice of nursing home (why a culturally profiled one?), and reason for transferring care from home to a residential nursing home. The interviews were mainly conducted by author MK and the interviewees chose the language to be used during the interview (either Persian or Azerbaijani, both of which the author MK is fluent in). The interviews lasted for about two hours and were audiotaped. They were then de-identified and transcribed verbatim to the language used during the interview. As authors LCH and EA are not fluent in these languages, the transcribed data was also translated into Swedish verbatim and controlled and corrected by an external researcher, who is native in Persian.

Data was then analyzed using the four steps of content analysis presented by Elo and Kyngäs (2008): (1) Repeated readings of the interviews and the creation of an overall picture by reading the interviews several times; (2) The data was organized by coding and then categorizing. An encoding is when a label is put on a phrase or a sentence. Categorization is when encodings with the same meaning are gathered under one common attribute but still contain nuances within and between the encodings (Weber 1990). This was done first by making an open coding, where relevant utterances were identified and highlighted. These were then coded by making headlines; (3) Codes were compared in terms of similarities and differences to be grouped, abstracted, and categorized into categories and subcategories; (4) Finally, the subcategories were grouped into the category they match with. In order to assure inter-rater reliability, the coding of the data was performed as follows: (I) All authors read all the interviews; (II) together, we developed a coding system; (III) one person coded; (IV) we all had joint discussions about these codings; (V) the specific problems that arose in coding were discussed by all.

### Ethical Considerations

The study has undergone ethical vetting by the Central Ethical Review Board (EPN), and has been approved (Dnr: 2012/180–31). All participants were given verbal and written information about the study and all persons included in the study has given their written consent.

## Results

Analyzed data from the 20 interviews resulted in identification of four categories and ten subcategories relevant to the studies aim of exploring family care giver’s decision to move their family members with dementia to culturally profiled nursing home in Sweden (Table 2).

**Table 2:** Overview of main result of categories and subcategories

	Categories	Subcategories
➔Transition process ➔	Pre-decision	Intra-familiar processes
		Lack of physical skill
		Lack of social constrains
		Need for professional care
	Decision	Finding and collecting information
		Excluding of the family member with dementia
	Transition	Strategies for transition
		Legal Restrictions
	New life	New life For family care givers
		New life For the family members with dementia

In the following, each category is presented as a time continuum of the transition process from home to nursing home and each subcategory described in the text.

## Pre-Decision

Central to the pre-decision phase was the assessment of the symptoms of cognitive challenges in the elderly relative with dementia by the persons implicit appointed the role as family care giver. As the symptoms became more manifest and the everyday life in the family became affected, the demand of care became clear to all of the informal family caregivers. As indicated in Table 1 this category is separated into subcategories having common differentiating characteristics within the category.

### Intra-Family Conflict

One pattern that emerged during the interviews was the cultural differences in terms of lifestyle. Taking care of the sick and needy parents is perceived as a traditional norm while at the same time the obligation to care within the family is decreasing among the interviewees. This was especially noticeable in the way the individual roles of the interviewees within the family were affected. The interviewees said as their individual roles were challenged, stress arose within each family member as they had difficulties in taking care of loved ones with dementia. This was due to the lifestyle they had created for themselves as well as the adaptation to traditional norms and expectations Amir (Male; 60 years old) described this situation in the following way:

**Interviewer:** You mentioned before that your mother lived every week at one of your families (brothers). Did that not create problems or did it not lead to tensions within their families?

**Amir:** If you like it or not, it becomes a problem and that was it. My wife is Iranian, she has her way of life and a way of life different from my mother and she wants to have a life with her children and her husband, not have someone else will come in and stay for long time in our home. Mothers circumstances did not fit into our lifestyles simply and we all showed complete understanding to her situation. I suggested that we should find a good/appropriate service home or nursing home for mom so she can get the help she needs by the staff.

Dissatisfaction in relationship is neither a new nor an uncommon problem. Which conflict strategies one use to deal with and finally what can help to create a good relation was up to each person’s strategy. When the desire to maintain traditional family structure cannot be realized, disappointment and coolness may play a role in the relationship that contributes to rejection and distance. Even language difficulties inevitably contributed to the withdrawal as evidenced in the following case. Bahman (male, age 49) was married to a European and had children. He was an office clerk in Iran and is a self-employed entrepreneur in Sweden. He and his brothers used to come to the nursing home to visit their mother.

**Bahman:** Honestly, it hurt me to see my mother come to us as a guest, she did not speak, she would eat a bit and it was just me hanging out with her, but it was not my wife's fault, but mother herself who should also make herself feel at home, she was welcome to us. Mom’s expectations, reticence and not being able to speak Swedish caused many misunderstandings.

The “aloofness” that was attributed to a lack of communication according to the interviewee, was a result of the language problem and presumably parental view around issues of marriage. However, another difficulty that led to the relative’s decision to move the family member with dementia to a residential care was that the mother’s life-world differed from the children’s. Being able to process the grief that eventually pained him, Bahman spoke warmly about a residential care that was an ideal residential care for the mother. That the mother could speak Persian language with the staffs and can have social intercourse by using common language in the nursing home gave him great joy.

**Bahman:** Here everybody speaks Persian and the food is Persian food. Mom cannot speak another language. Mom and I understand what the doctor, the staff and nurses say. I’m divorced, but that was not all because of mom, there were other factors that influenced the divorce.

Often, people with immigrant backgrounds are portrayed to already have a traditional care provided for them within the family, but this traditional care home is not always guaranteed or obvious. Due to the new, different, and foreign lifestyles that the informants are in, they use the option of a nursing home as a possibly.

Ziba (female, age 40) is an informant who tackles a different aspect of the problem that affected her decision to move her mother to a residential care home. Severe stress and high demands affected the family due to the mother who has dementia; but also other family matters added to this stress (a child with ADHD).

**Ziba:** When mom was at home I had to do exactly what she demanded. I have had no social life just because of mom. Mom made the life very hard for me. And she was very harsh on me.

The harshness of the caregiver’s situation affected both the personal freedom as well as the social atmosphere inside the family. Some participants even expressed it as a crack in the marriage leading to a divorce as in the case with Ziba. She claims that the underlying cause of her divorce was her mother’s situation and behavior. It was not always easy for Ziba to avoid hearing the coarse language of the person with dementia, her mother. Although the separation was not the only solution, Ziba prioritized her mother before her marriage. She finally found that the problem remained unsolved and decided to move the mother to a residential care home.

**Ziba:** My divorce was solely due to my mother and taking care of her at home. The language my mother used against my husband and my daughter angered them in the highest degree. Finally, it became impossible for me to reassure all those at home and should be doing my priorities. I prioritized my mother and daughter, but the problem was not solved as I had imagined.

She continues and says that the mother deliberately violated her son in law, and he therefore disapproved collaborating with his wife in giving care to his mother in law.

**Ziba:** My mother did things and used very bitter and foul language against us at home, so my husband did not help and only I took care of her. Mom deliberately humiliated him and then said that she did not mean anything and I was tired of being a peace mediator between them hence we parted. I am more than 100% certain that it was my mother who caused the divorce.

### Lack of Physical Skill and Lack of Social Constraints

The impact of family members’ situation in the care of a family member may vary. Seeing a beloved family member with dementia creates feelings of grief, regardless what age they themselves may be in. Being able to take care of the person with dementia requires both physical and mental demands on the caregiver, especially if the person with dementia is a family member. The interviewee Dana (female, age 49) said:

**Dana:** I myself became depressed after losing my mother and now my father is as ill. I’m tired and cannot cope with extra problems. In the beginning my father began to behave differently and became more and more forgetful, I become his home care assistant, personal assistant, although, I myself was neither physically nor mentally a healthy person, but I tried to help him. Then I asked the municipality for help to send someone to come to my father’s home and clean his apartment. After a long time, the memory clinic called my father to reexamine him. I was very ill and was not able to drive my father to the memory clinic for consultation and father went alone or together with a home care assistant.

Another female informant describes her situation in a different way. Being able to take care of someone with dementia also requires physical strength, in order to clean the house, washing, ironing, cooking, shopping, etc. at the same time she should take care of the rest of her family members; the children, her husband, the home and even to take care of herself was too much for her to handle.

**Hana (**female, age 45): I understood taking care of mom all of these years was not an easy job at all. Because I have had a herniated disc in my neck and couldn’t help her. I got help from the municipality for cleaning mom's apartment, I noticed that my hands would go numb, finally, I became ill. I could not help myself or mother any longer.

To care for an immediate family member with dementia, especially someone whose former personality and former managing ability of different situations in life has changed, can lead to huge feelings of loss, frustration for the family members, and as a consequence the person with dementia can be portrayed as a stranger to them and even to the person with dementia. In addition to the physical impairments, delusions often contribute to the otherness and also distance themselves from close loved ones around them. It is this behavior which allows even relatives to consider him or her as an outsider and even as a “demented other” (Naue and Kroll 2009).

**Sherwin (**female, age 50)**:** If I may briefly summarize mother's behavior, I certainly do not call mom for a ‘human being’. None of us dared to leave mom alone in her apartment.

In another case there arose a responsibility on a mother (Ziba) besides just taking care of her child. The mother’s (Ziba) own mother was diagnosed with dementia requiring her to become a caregiving mother, giving care not only to her mother with dementia, but also to her child. The time and effort required for her role as a caregiving mother was very physically and mentally demanding. The caregiving mother even said, “The whole situation would have become intolerable.” Having two needy individuals at home became difficult for the caregiving mother who told me that her mother’s maternal behavior exacerbated her life and it pained her. Finally, the caregiving mother chose to move her mother with dementia away from home to a residential care home.

**Ziba** (female, age 40)**:** It is impossible for me to take care of my mother at home with my child who has serious health problems. Mom and my daughter do not get along with each other and this makes me sad and down. My daughter has ADHD and she cannot cope with the extra stress my mother caused and causes when she is home with me. If my daughter and my mother were at home the whole situation would have become untenable.

### Need of Professional Care

To care for a family member with dementia is an excessive challenge for family caregivers. Sometimes care for the person with dementia requires the caregiver’s full attention and schedule. Certain mobile healthcare routines improve the safety for people with dementia. One way to handle the situation is to use various technical alarms – as for their safety functions as a surveillance system. For a few years, Hana has been taking care of her mother without her sibling’s assistance. She, like many of the people interviewed, was employed by the municipality to be her mother’s caregiver. After using different strategies to teach her mother with dementia not to call her in different inopportune times, she met another face of reality, namely that the mother with dementia had disappeared. Then she considered that her mother’s need of help is out of her ability and capacity. She says:

**Hana:** Mom had the alarm, but she used the alarm unnecessarily all the time. After each alarm we came to her and realized that it was a false alarm. Can you imagine it was a lot of work to run to her unnecessarily? It also often would happen in the middle of the night, she would call at two o’clock in the morning to ask if it’s two o’clock in the day or in the night. I gave up and told her that she could not call us at night. Then she rang at four am and wanted to speak with my children. Finally, I decided to turn off the phone. Then one morning, I received a phone call (emergency) that mother had pushed the alarm and she was in the hospital and we should drive there to pick her up. So I did it. One day I came home to give her medicine, I found her not at home. Mom was missing.

To sum up, in the beginning, the decision to care for the family member with dementia was made by his or her own loved ones out of their own concerns. As they attempted to care for their loved ones on their own, different challenges arose: intra-family conflicts, lack of physical skill and lack of social constraints, and finally finding themselves in need of professional care.

## Decisions

The next category in the transition process is the decision making. Included in the category are the 2 subcategories; finding/collecting information and excluding the family member with dementia. Almost everyone interviewed initially cared for their family member with dementia at home, but as the dementia progresses and the ensuing crisis the family faces intensifies, the family becomes faced with the inevitable decision of admitting their family member with dementia into a nursing home. Most family members felt they had no other option than to move the person with dementia into a residential care facility and almost none of them discussed their decision with the person with dementia. This could be compared with what Hulko & Stern (drawing on both Chan 2004 and Kagawa-Singer and Blackhall 2001) has previously reported, namely that *“within other cultures, decision-making is primarily seen to be the duty of the family as a form of obligation and protection, regardless of an individual’s cognitive status”* (Hulko and Stern 2009:82). Also, family members’ positive attitude towards nursing homes and their care giving made it easier for them to make the decision of admitting their family member into the nursing home. Of all the interviewees, nobody felt guilty for the decisions they made. Most felt that they had done everything they could for the person’s wellbeing. Some families even expressed relief when the caring for relatives with dementia at home had ceased and they felt no regret over the decision. This could be compared to what Adair (2007) has described concerning how decision-making is about deciding what action to take, usually involving a choice between options.

### To Find and Collect Information

Family caregivers used what social networks they had to help them acquire information regarding residential care. Information and access to family support have been different with different individuals among my interviewees. Information varied from individual to individual. One had turned to his circle of friends, another to the shop nearby, another turned to the local radio broadcasts, and another went to social services. This is what Sherwin said on her attempts to find a nursing home for her mother.

**Sherwin** (female, 50): I have a good friend who worked in [name of work place] as social worker. I called her and told her about mother’s situation and asked her if there are any nursing homes or service houses where my mom could stay. She suggested that I should apply for home care services to help mom and she renounced all forms of nursing homes. I explained that we had home care service and even my own brother lived with mother in order to help her and take care of her, but it is not working any longer and will not work anymore. Then she suggested Anahita that has recently opened for business. She spoke very positively about the Anahita. She gave me the phone number of the nursing home Anahita and the day after I called and two days after we moved mom to Anahita. By that time, Anahita had few staff services at the resident home. There was a nurse, the manager, and two or three other ordinary staff.

Helen was a teacher in Iran and had worked in a library in Sweden. She had lived and taken care of her mother for quite some time. Helen collected information from a person with previous experience about residential home care. This person had a positive attitude toward a certain nursing home and recommended it to the interviewee that helped the interviewee to make her own decision quickly. This recommendation gave Helen significant help to make her decision:

**Helen** (female, 60): I talked to an Iranian who has a supermarket in the area in which we live. His own mother had lived there and last January she passed away. He recommended the same nursing home in which his mother was placed.

The contact with the elderly care home or knowledge about the nursing home was probably inadequate. As the emerging need to approach an adequate nursing home arose, information seeking was noticeably harder to obtain. After getting to know about the nursing home through the Iranian radio broadcast, Ziba talked to different authorities about proceeding with the formal issue, one authority she approached was even the manger. After meeting with the required authorities, she got approval to move her mother with dementia to the sought after nursing home. She highlights the importance of the nursing home as having saved her life.

**Ziba:** I had no one to help me. One day I heard on the news [on Iranian local radio broadcast] about [name of nursing home] nursing home and I called them immediately. After talking to different authorities, and even to the nursing home care manager, decided to move there. The nursing home has saved my life.

A high level of motivation increases the attention paid by the individual to relevant information and the comprehension of such about the nursing home. But not everyone knew where to turn. This interviewee said:

**Interviewer:** Did you have no one to help you?

**Hana:** No. But it was the only municipality that helped me in the beginning by employing me as my mother’s caregiver. Otherwise no help from anywhere /anyone and I became empty inside (my strength gone). Nobody helped me, no. I did not know where I simply could leave my mom and with whom I couldleave her. I asked the municipality for help and they asked me to talk with the doctor. Doctors did help me to move mother to this nursing home.

### Excludes the Family Member with Dementia

There were no reciprocal understandings or agreements. The family member with dementia was almost always excluded from the decision to be moved to a nursing home. Following examples shows how nobody asked of the persons with dementias own opinion of staying or leaving the home:

**Interviewer:** You moved your mother here against her will as you said earlier… How did that happen?

**Sherwin:** Yes that’s right. She refused to come out of her apartment. However, we explained to her that she could not go on living in that apartment…(Sherwin moved her mother to the nursing home with her mother’s consent and explained her mother’s feeling about it)…In the beginning it was very hard for her t o stay there. She asked us to move her away.

**Interviewer:** Did you ask your mother if she wanted to leave her home and come to the nursing home?

**Helen:** I have decided to give me a chance to live together with my children. I hope that my new life, now that mother has come to the nursing home, to become a normal life without complications. It was not good for mother to be at home with me because Mom was a prisoner and I was the prison guard … Can you imagine that?

**Interviewer:** Who decided for your mother to come to the nursing home? Did your mother decide that she didn’t want to be a burden?

**Aylin:** I couldn’t help Mom any longer. I talked to my brother, who lived abroad, and he came to me and we discussed and decided the best thing we can do is to transfer our Mother to the nursing home. We are very satisfied and happy that mother is here. (Even though the interviewees mother didn’t want to move to the nursing home.)

Talking about autonomy is essential if focus is on self-determination, i.e., to obtain the decisions concerning one’s own life based on the desires one might have for how he/she wants to live their life. At the same time, even with limited agency, one can choose to live their life, just as one would like, and this is perhaps eve truer when one deals with dementia.

After conflicts and challenges, the family caregiver eventually came to the conclusion that his/her current life-style was not working, and they made the decision to move their family member with dementia to a nursing home. After the care giver had come to the conclusion that a nursing home was the best place for residency for their loved one with dementia, they began acquiring information about nursing homes as they attempted to find the most suitable one. Each caregiver used his/her own different strategies to obtain the information needed. However, even if family care givers often did not consider the opinion of their loved one with dementia the decision was almost always described as out of concern for the well-being for the person with dementia.

**Ayeh:** (when talking in the interview about the fact that her mother was raped) Some male neighbors (they were not aware she was diagnosed with dementia) mistook my mother’s attitude towards them and misused my mother. Now that she is at a nursing home she is safe. No one could hurt her.

Ziba said similar that in case something happened to her mother, someone from the nursing home would be able to take care of her and that she would be able to escape any accusations from relatives, in the event that something bad happened.

Thus, they were focused on making sure their loved one was moved to a safe environment, so the overall lifestyle of both themselves and the person with dementia could improve as quickly as possible. Because of this, the opinion of the family member with dementia often went unheard. The family care-givers were generally not excluding their family member with dementia out of carelessness or lack of concern but were rather focused on the well-being of the person with dementia.

## Transition from Home to Nursing Home

After the relatives receive guidance and information about the nursing home they wish to move their family member with dementia immediately to the nursing home. Almost all of the families were aware that their relatives would receive a form of health care that would be advantageous to the person with dementia. Despite the decision, none of the families abandoned their family member with dementia at the nursing home. The families were still interested and willing to be involved with the care given at the nursing home.

Almost all people with dementia who have moved to a nursing home came from their own living arrangements but some also came from a previous care unit where care wasn’t sufficient for them. Because the previous living arrangements were not sufficient or even unsafe due to their condition, the move to the new nursing home was of great priority. After all, it was not easy moving to the nursing home for them. While the family member with dementia had a hard time moving, some of the families decided to get creative and use different strategies in order to persuade them to move. These strategies were based on falsehood and loving deception.

**Hana:** In the beginning, she had hell here. She wept and beat herself all the time. Ever since she has arrived, she wanted to move back home. I lied and said, Mom, I bought this place for you and this is your own. Even today, she thinks that the condo where she lives is purchased by her.

Despite all, the focus was entirely on living at their previous home for those relatives with dementia. Not all relatives with dementia had wished to move to a nursing home, but there were some who had difficulty to accept the new living quarters as their new home.

**Ziba:** Mom cried and said she does not want to move from my home, but it was impossible for me to take her home with me. Having mom with me, life would be very difficult both for me and for my daughter. I promised her if she moves to nursing home I will be with her every day. It was not likely that I would be with her every day, but you could say that I was cheating on her just because she will be moving from me … It’s not hard to fool mother, it may be possible to trick her. I told her that she could come to me and I can be with her quite often. I told her that I do not have extra room for her here at home. Three people living in a two-room apartment can be crowded and difficult. I promised her that if I buy a larger apartment she can move in with. I tried my best to convince her otherwise, she did not want to move to the nursing home.

Some people with dementia want to go home, back to Iran, so family members would take them to culturally profiled nursing homes, which would have a room that resembled the home of their loved one with dementia, and say to them, “Now we are home in Iran.” This would be an effort of lovingly deceiving their family member to better conditions.

### Legal Restrictions: Geographic and Socio-Economic Problems

One of the many issues that these families deal with when their family member moves to a new nursing home is the geographical distance between them and the new nursing home but also the rules within the geographical area they live in. The families that live within the certain municipality/district boundaries have to deal with the respected municipalities rules. These rules complicate the process of becoming enrolled in the new nursing home. They are required to meet with various responsible authorities in order to receive permission to move into the chosen nursing home that often takes a longer time than one had anticipated

**Helen:** [The A] municipality refused to pay for [the B] municipality. I spoke with the doctor who in turn spoke with different authorities and nursing homes. After a long struggle they said that there is a room available, but the [the A] municipality said no. After the municipality gave us a positive answer there were no available rooms at the nursing home and we should wait again. It was not so easy at all.

Transition is the process of moving and adapting to the new environment and life-style for the person with dementia. The first part of the transition process is relocation often with three phases from being overwhelmed, the person being forced to adapt and his/her initial adjustment to the situation, the final adjusted to the new situation (Kralik et al. 2006). Moving the person with dementia to a nursing home as quickly as possible was a high priority for the family care givers, due to the insufficiency and danger of the current living situation. The person with dementia wanted to stay in their home, so the family care givers would use strategies to convince or deceive him/her. Another challenge family care givers have to deal with when moving their family member with dementia to a nursing home is legal restrictions, i.e. specific rules or guidelines that are set for their individual municipality.

## New Life for Family Care Givers and a New Life for the Family Members with Dementia

Care in the home involves demands, stress, and sometimes havoc. However, no matter how long prior to placement the relative has cared for his family member with dementia, they felt satisfied after placement. Once the care in the family home ceased, the family caregivers found more time for themselves in order to pursue social and personal interests. The family caregivers realized that even though they love their relative that deals with dementia, they were also a burden. The former family care givers voice their dilemma of prioritizing their relative with dementia or their immediate family.

**Interviewer:** Your mother is here at a nursing home. How happy and satisfied are you with your life now if I may ask you?

**Ziba:** The best thing that has happened to me is that I have enough free time for myself and I can spend some more time with my daughter, than when Mom was here at my house. We have the freedom to feel at home in our own home/house, which was previously occupied by my mother. A nursing home is the best place for mother and has saved us and our lives. None of us can cope with a parent who is so demented.

Many family care givers had a pre-planned lifestyle, because care is required around the clock, day-and-night, and the careers’ own needs had to be put aside. Many stated they felt liberated and even relieved after placement. An opportunity to delegate time toward other areas of life was highly anticipated. The interviewee desired to spend more time with her children.

**Helen:** I have personally decided to give myself a chance to live a life with my children. I hope that my new life after my mother has moved to a nursing home will be a normal life without complications.

When a family caregiver was not able to be with their relative with dementia, they often experienced much concern for the well-being of their relative with dementia. According to another interviewee (Ayeh), the transition to a secure residential care was an indescribable, joyful, and momentous occasion. These positive feelings were due to the fact that she felt that her mother’s nursing home was reliable, secure and safe. She was able to find time with for herself and could sleep at night without worry because she knew her mother’s care was remarkable.

**Ayeh** (female, 40): Honestly, I’m happy now. The worst period with mom is over. If I had wings, I could fly with joy. I feel free and independent. Now I can sleep quite easily. I did not have normal sleep. Often I would awake in the middle of the night and wondered what mom is doing now? Because mother is a smoker, she had burned the furniture and one day the whole house was in flames. I must say that we've experienced horrible things with our mom. Now when I’m sitting in front of you and telling some episodes of my mother's life, I get the feeling that it was a bad dream (nightmare I had). Now I know that I will not see the “awful movie”, “the nightmare” anymore. I'm not afraid anymore, but still it's sad after all.

Another interviewee described the positive attitude and belief that the interviewee had towards the care her mother received she compared with that of a comfortable, luxurious hotel. A place where all of her mother’s needs would be met. This is Ester’s (female, 60) version:

**Ester:** The nursing home is like a hotel. Mom takes a shower almost every day in her own bathroom. She has her own kitchen and bed, which is the best that one can lay on [the nursing home used to arrange the bed]. Each night the staff will go into mom’s room and see if she’s asleep or awake. They have Iranian television, Iranian food. The nursing home is like a hotel not a residential care. When mother calls them, within a few seconds the staff come to her and helps her.

There was another interviewee (Ziba) who expressed joy of avoiding false accusations from distant family members, along with the assurance that her mother is receiving adequate care. She stated that it is possible if her mother sustained an injury under her care, family members living in different countries may accuse her of carelessness towards her mother’s condition. She said:

**Ziba:** Despite everything, I am happy for my mother because she has access to medical assistance and the staff checks on her even at night time. The staff in the department can help her immediately. If something happens to her no one will criticize me for leaving mom home alone or if she falls down no one will accuse me for not have noticed or something like that ... she is there and the staff takes care of her ... it feels really nice to not be constantly worried.

Based on Bahman’s personal understanding and previous experiences he had with healthcare in Iran, he described the nursing home for his mother’s well-being:

**Mahin:** What did you want to do for your mom that you have not been able to do for her in Sweden?

**Bahman:** The best thing my brothers and I have done for our mother is that we have placed her at this nursing home. Honestly doctor (interviewer laughs)- we knew what would have happened to mom if she were in Iran or in a hospital in Iran.

Overall, the family care givers all showed extreme satisfaction for the new lifestyle they enjoy with their loved ones now in nursing homes. Their once stressful lives seem to be vanishing, as they now have the opportunity to focus on their family and other social interests. One interviewee even compared the time of her life she cared for her mother with dementia as a nightmare. She had to deal with a lot of traumatizing events. One in particular was when her mother had set the house in flames, while smoking. Many family care givers dealt with social pressure, as they could potentially be blamed by other relatives if any harm would occur to their loved one with dementia, while under their care. They are now able to live without these anxieties and constant worries for the well-being of their family member with dementia. They have been put at ease knowing the exceptional care their family members with dementia are now receiving. One interviewee even positively compared her mother’s nursing home to a luxurious hotel. She mentioned how her mother has her own bathroom, kitchen, and bed. She even enjoys the comfort of her own culture, as she is fed Iranian food and as they show Iranian television. The staff provides constant care and is there to assist her in just seconds, when she is in need.

## Discussion

The aim of the study was to describe the experience of Iranian immigrants living in Sweden whom have been caring for relatives with diagnosed dementia diseases and the reason for the transition of relatives with dementia disease to the nursing home. In the beginning, the decision to care for the family member with dementia diseases on ones own was made out of concern for the person with dementia disease, one wished to help them in all ways possible. However, as they attempted to care for their loved ones on their own, different challenges arose. Of course, the interviewees have different experiences from the period in which they were caregivers. Multiple difficulties led the interviewees to make the decision to transfer their beloved relatives with dementia to a (culturally profiled) nursing home. Sometimes, the difficulty that led to the relative’s decision to move the family member with dementia to a residential care was due to the person with dementias life-world, which differed from their children and others in the surrounding area, causing difficulties for families to cope. This agrees with Mc Grews and Hirschfeld’s (1979) argument that a high capacity from the caregiver is required. Physical and social problems were common occurrences for the caregivers, due to the exhausting tasks of caring for their family members. Along with this was the lack of both informal and formal support in the beginning. Most of the interviewees had no access to informal support, which was mainly due to the fact that the greater family, after migration, had been spread in different countries, but partly also because of the difficulty in getting along with each other. Therefore, causing them to be alone giving care, with no assistance, and with little knowledge about support-groups. Many of the interviewees give this as a main reason to why caregiving at home needed to end.

The findings also suggest that the fact that it is an *ethnoculturally profiled residential care home* is crucial in the making of the decision to cease care giving at home. To some extent a profiled nursing home seems to ease the guilt of not fulfilling ones duty of filial piety (for further discussion, see Antelius and Kiwi 2015) and making sure that mother and father is taken care of by someone who speaks the same mother tongue and have similar cultural background. It feels safe and secure. However, it must also be understood in relation to the fact that the person with dementia has not been allowed to be part of the decision to move to the nursing home. Several of the interviewees discuss the fact that they “lovingly deceive” their relatives for their own good, so that they can have the best care possible, something the relatives have become too tired, or are too untrained, to give. The choosing of an ethnoculturally profiled nursing home is perceived as help in such “deception” as the person with dementia could be told (and often believes) that they are indeed back in Iran.

Mainly however, the one thing that this study shows more than anything, is that the family caregivers experienced great (personal) difficulties in providing care at home. They had nightmares, they were exhausted, they had no time for their own children and other social activities. One did not dare to leave the house, and if one did they constantly worried about person with dementia being left alone. Thus, many interviewees agreed that when they had transferred their loved ones to a nursing home, they became relieved of the constant stresses of worrying for the person with dementia, at the same time as they state that they themselves felt less “tied down.” Thus, the decision, and the result of the move, is understood as beneficial for all parts, both the person with dementia and the family caregiver, at least according to the former caregivers’ opinion.^4^ The constant anxiety and worrying for their loved ones has gone away. They are still able to provide for their loved ones; they frequently visit the nursing home and help their mothers and fathers (or sometimes husbands and wives) with many things. But, the main responsibility has gone and almost all of them express a sense of relief to be able to live “ones own life”. This of course could be discussed from many different perspectives, however perhaps the most important one is that during the recent decade(s) there has been a growing recognition that people cannot be assumed to be incapable of making decisions about their own care just because they have a dementia diagnosis (Kitwood 1997). Thus, the formal right for people with dementia to take part in decisions about their own care has been strengthen, by legislation and international agreements. This we argue is of course important to recognize, that persons with dementia have both the ability – and formal right – to voice their own feelings and preferences (see for instance Bartlett and O’Connor 2010; Boyle 2008). We do *not* wish to argue against that. However, we find it relevant to also discuss what it is then that makes relatives take decisions *for* the person with dementia.

As a recent review just asked for – more research regarding how care decisions are made and experienced *in practice* (see Taghizadeh Larsson and Österholm 2014) – this paper wishes to illuminate how such decisions are made, and experienced. And our study show that in these particular cases the person with dementia is clearly still left out from the decision. The family caregivers all state that they are too tired to continue care at home, the move to a nursing home is a must. However, at the same time they know that their loved ones with dementia do not want to move. So, they leave them out of the decision. How this is discussed and dealt with at the formal assessment meeting with a social worker (which is required in order to be able to move to a nursing home in Sweden), this study does not reveal. We do not know if the persons with dementia disease are present – or if present, that they know that their inclusion matters – at these formal meetings. What we do know from this study is that the family caregivers all state that they cannot continue caregiving at home, so they “lovingly deceive” them. One could argue if this is right, or if it is wrong. But that would not be all that productive. Because as this study shows, this is how decision are made, and experienced in practice. We must then understand why this is still so. And one key pattern that emerged during the interviews and which has been showed in the excerpts above, is that the cultural differences in terms of lifestyle are changing. Although it goes beyond the scope of this particular study to compare between cultures and/or countries different long-term care systems it is worth mentioning this result of changes in lifestyle as having an affect upon cultural values in relation to filial piety in relation to other contexts. A recent Iranian study (see Mortazavi et al. 2015; Peyrovi et al. 2012) has showed that although also the Iranian society is experiencing an aging population, family members decisions to utilize formal care remains challenging as ‘Iranian culture is persistence to the transfer of elderly people to nursing homes’ (Peyrovi et al. 2012:185). Our study thus suggests that taking care of sick and needing parents is still perceived as an Iranian traditional norm, while at the same time the obligation to provide this care within the family is decreasing among the interviewees who have lived for quite a substantial amount of time in Sweden. It might seem harsh, but as Amir put it: *Mothers circumstances did not fit into our lifestyles*.

### Limitations of the Study

At the time of the study, only one nursing home for Persian-speaking individuals living with dementia existed in Sweden, thus one possible limitation is that the study was conducted on a convenient sample of all relatives connected to the residents of this particular residential facility. Possibly it could prove beneficial to investigate differences between residential care homes with different cultural profiles as well as differences between responses of caregivers from the same cultural background but in different contexts.

## Appendix

### Interview guide

- Would you like to tell about yourself, how old you are and how long you have lived in Sweden, what is your profession, family status, and what was your occupation in Iran.
  Who took the decision for the parents to move to Sweden, and why? What was their profession in Iran and how was their health before they moved to Sweden? Did your parents live in their own home or did you live with them?
- Does your family (father / mother) live in their apartment in Sweden or live with you and your family. Why/why not?
- Who takes care of the parents? How long have you taken care of your parents? Did the rest of your family cooperate with you and why/why not? How has your social and family life functioned during the days you have been caregiver?
- Did your Parents receive home help service? How long? Why/why not?
- How did your father / mother or your husband / wife feel before you brought him / her to the doctor? Was the person diagnosed with dementia, did you understand what the diagnosis was about and did you know what dementia was and did the doctor explained what the diagnosis is all about?
- Did you feel lonely and sad; with whom did you share your loneliness and sorrow?
- Who made the decision to move family member with dementia to the cultural profiled residential care? Why? What was other family member’s attitude toward the decision?
- Did they want to move to the culturally profiled nursing home, what did you tell them and how and in what way convinced or persuade them to move? How did you collect information about this culturally profiled nursing home? Who helped you to get in touch with just this residential care and what does your family in Iran and outside Iran says? How does it feel to expose the truth that dad/mom has been diagnosed with dementia and he/she lives in nursing home? Do you feel remorse and awkward about it? Why/why not?
- Have the parents / spouses been staying in a short term accommodation or in a Swedish-speaking residential care before? Why did you move him / her to this nursing home?
- What are the advantages and disadvantages of this residential care and how does it feel to move the family member here? How are you and your other family members feeling now that mom / dad, husband/wife lives at the culturally profiled nursing home?
- How long have you been taking care of him/her and how your life has changed for the better after the father’s / mother’s/ spouses moved to residential care? What makes you happy or sad to see the family here in the nursing home? Why/why not?
- How often do you visit your relatives here in nursing home, do you spend a day and night here with him/her now and then, why/why not?
- What does the family/ spouses say about the life here in this nursing home? What makes them happy or the more or less disappointed from your point of view and why? Do they want to move from here to another place, why/why not?
- If Mom / Dad had been in Iran, would their life have been better for them and easier for you here?

## Footnotes

1 It should be noted that this is one study out of several in a comprehensive research project (*Ethnocultural Diversity and Dementia Disease)* conducted at Centre for Dementia Research (CEDER: www. http://www.isv.liu.se/ceder?l=en), so other perspectives, such as for instance staff members’ perspective on caring for the individuals with dementia diseases, or the resident’s perspective of being in a nursing home will be explored in other papers. It should also be noted that, even though this is not the focus of this paper, in Sweden, one has to have a formal decision on care service (as decided in assessment meetings with social workers) in order to be able to move into a nursing home. For a thorough review on how these decisions are made and experienced, see Taghizadeh Larsson and Österholm 2014.

2 Management and planning of care is split among three authorities; central government, city councils and local authorities of which all is represented by elected political bodies which have the right to finance their activities by levying taxes and fees within the frameworks set by the Social Service Act. Determined by an assement of needs, anyone with a permanent residency in Sweden is eligeble for (elderly) care. To guarantee that all elderly in need of care are able to recieve such, and to avoid financial exploatation, a maximum monthly fee for long-term care is set by central authorites (Fukushima et al. 2010).

3 All names/terms, such as for instance ‘Oriental’ are the nursing facilities own.

4 See Kiwi and Antelius forthcoming *,* for a discussion on the person with dementias own views.

## References

[CR1] Abdollahpour I, Noorozian M, Nedjat S, Majdzadeh R (2012). Caregiver burden and its determinants among the family members of patients with dementia in Iran. International Journal of Preventive medicine.

[CR2] Adair, J. E. (2007). *Decision making and problem solving strategies* (Vol. 9). Kogan Page Publishers.

[CR3] Antelius E, Kiwi M (2015). Frankly, none of us know what dementia is: dementia caregiving among Iranian immigrants living in Sweden. Care Management Journals.

[CR4] Bartlett R, O’Connor D (2010). Broadening the dementia debate: toward social citizenship.

[CR5] Boyle G (2008). The mental capacity act 2005: promoting the citizenship of people with dementia?. Health and Social Care in the Community.

[CR6] Castles S (2002). Migration and community formation under conditions of globalization. International Migration Review.

[CR7] Chan HM (2004). Sharing death and dying: advanced directives, autonomy and the family. Bioethics.

[CR8] Elo S, Kyngäs H (2008). The qualitative content analysis process. Journal of Advanced Nursing.

[CR9] Emami A (2000). "We are deaf, though we hear; we are dumb, though we talk; we are blind, though we see": understanding Iranian late-in-life immigrants' perceptions and experiences of health, illness and culturally appropriate care.

[CR10] Emami, A., & Ekman, S. L. (1998). Living in a foreign country in old age - life in Sweden as exerienced by elderly Iranian immigrants. *Health care in later life, 3*, 183–198.

[CR11] Emami A, Torres S (2005). Making sense of illness: late-in-life migration as point of departure for elderly Iranian immigrants’ explanatory models of illness. Journal of Immigrant Health.

[CR12] Ferri CP, Prince M, Brayne C, Brodaty H, Fratiglioni L, Ganguli M (2006). Global prevalence of dementia: a Delphi consensus study. The Lancet.

[CR13] Fukushima, N., Adami, J., & Palme, M. (2010). *The long-term care system for the elderly in Sweden*. European Network of Economic Policy Research Institutes (ENERPI) report no.89. Available for free downloading at: www.ceps.eu

[CR14] Hajighasemi, F. (1994). *Invandring på gamla da'r: sextio äldre iranier berättar*. [Immigration in one’s elderly days: sixty older Iranians narrate] FoU-byrån, Stockholms socialtjänst.

[CR15] Heravi-Karimooi M, Anoosheh M, Foroughan M, Sheykhi MT, Hajizadeh E (2010). Understanding loneliness in the lived experiences of Iranian elders. Scandinavian Journal of Caring Sciences.

[CR16] Hirschfeld, M. J. (1979). Families living with senile brain disease (Doctoral dissertation, ProQuest Information & Learning).

[CR17] Hirschfeld M (2003). Home care versus institutionalization: family caregiving and senile brain disease. International Journal of Nursing Studies.

[CR18] Hulko W, Stern L, O’Connor D, Purves B (2009). Cultural safety, decision-making and dementia. Troubling notions of autonomy and personhood. Decision-making, personhood and dementia. Exploring the interface.

[CR19] Kagawa-Singer M, Blackhall LJ (2001). Negotiating cross-cultural issues at the end-of-life: “you gotta go where he lives”. Journal of the American Medical Association.

[CR20] Kitwood, T. (1997). *Dementia reconsidered. The person comes first*. Philadelphia: Open University Press.

[CR21] Kiwi, M. & Antelius, E. (forthcoming). Ethnoculturally profiled residential dementia care: away from home, or returning home?

[CR22] Kralik D, Visentin K, Loon A (2006). Transition: a literature review. Journal of Advanced Nursing.

[CR23] Luppaa M, Luck T, Brähler E, König H, Riedel-Heller SG (2008). Prediction of institutionalisation in dementia. Dementia and Geriatric Cognitive Disorders.

[CR24] McGrew, K. B. (1995). Caregiving paths, patterns, and perspectives. Scripps Gerontology Center, Miami University.

[CR25] Mortazavi H, Peyrovi H, Joolaee S (2015). How do family caregivers of older people give up caregiving?. International Journal of Community Based Nursing and Midwifery.

[CR26] Naue U, Kroll T (2009). ‘The demented other’: identity and difference in dementia. Nursing Philosophy.

[CR27] Ngo, J. & Holroyd-Leduc, J. M. (2014). Systematic review of recent dementia practice guidelines. *Age and ageing*, afu143. 10.1093/ageing/afu143 25341676

[CR28] Omeri A (1997). Culture care of Iranian immigrants in new South Wales, Australia: sharing transcultural nursing knowledge. Journal of Transcultural Nursing.

[CR29] Peyrovi H, Morazavi H, Joolaee S (2012). Challenges of Iranian family members in decision making to put elderly people with chronic diseases in nursing homes: a qualitative study. Archives des Sciences.

[CR30] Priebe S, Sandhu S, Dias S, Gaddini A, Greacen T, Ioannidis E (2011). Good practice in health care for migrants: views and experiences of care professionals in 16 European countries. BMC Public Health.

[CR31] Riahi ME. (2008) Comparative study of the position and status of elderly people in the past and contemporary societies. *Iranian Journal of Ageing*, *3*(3&4):10-21. (Persian)

[CR32] SCB (Statistics Sweden) (2016). Utrikes födda i riket efter födelseland, ålder och kön. År 2000–2015 [Foreign born within the kingdom according to place of birth, age and gender. Year 2000–2015]. http://www.statistikdatabasen.scb.se/pxweb/sv/ssd/START__BE__BE0101__BE0101E/UtrikesFoddaR/?rxid=4c1437f3-0ae2-4028-af30-4f6285298f7a, retrieved 2017–01-18, at 11:40am.

[CR33] Söderberg M, Ståhl A, Emilsson UM (2013). Independence as stigmatizing value for older people considering relocation to a residential home. European Journal of Social Work.

[CR34] SoS (The Swedish National Board of Health and Welfare) (2015). Tillståndet och utvecklingen inom hälso- och sjukvård och socialtjänst [The state and development within health care and social services]. Report available for downloading at: http://www.socialstyrelsen.se/Lists/Artikelkatalog/Attachments/19747/2015-2-51.pdf

[CR35] Taghizadeh Larsson A, Österholm JH (2014). How are decisions on care services for people with dementia made and experienced? A systematic review and qualitative synthesis of recent empirical findings. International Psychogeriatrics.

[CR36] Torres, S. (2001). Understandings of successful ageing in the context of migration: the case of Iranian immigrants in Sweden. *Ageing and Society, 21*(03), 333–355.

[CR37] Torres, S. (2006). Different ways of understanding the construct of ‘successful aging’: Iranian immigrants speak about what aging well means to them. *Journal of Cross-Cultural Gerontology, 21*(1–2), 1–23. 10.1007/s10823-006-9017-z 17106646

[CR38] Weber, R. P. (Ed.). (1990). *Basic content analysis* (No. 49). Sage.

